# Supplementary material for: Identification of photoperiod-induced specific miRNAs in the adrenal glands of Sunite sheep (Ovis aries)
Source: Front Vet Sci. 2022 Jul 22;9:888207. doi: 10.3389/fvets.2022.888207 (PMC9354845; doi:10.3389/fvets.2022.888207)
Supplement: Supplementary file 1 [file Data_Sheet_1.ZIP › Supplementary file/miRDeep2 file.zip!/miRDeep2 file/Novel_Structure/LP42A3/Novel_187.pdf]

Provisional ID	:	NC_019475.2_18885
Score total	:	101.1
Score for star read(s)	:	3.9
Score for read counts	:	93.4
Score for mfe	:	2.2
Score for randfold	:	1.6
Score for cons. seed	:	
Total read count	:	195
Mature read count	:	183
Loop read count	:	0
Star read count	:	12

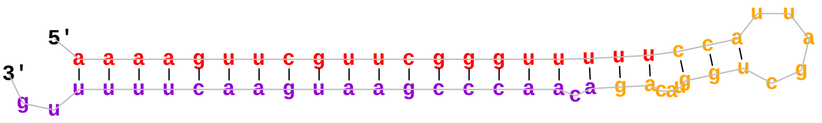

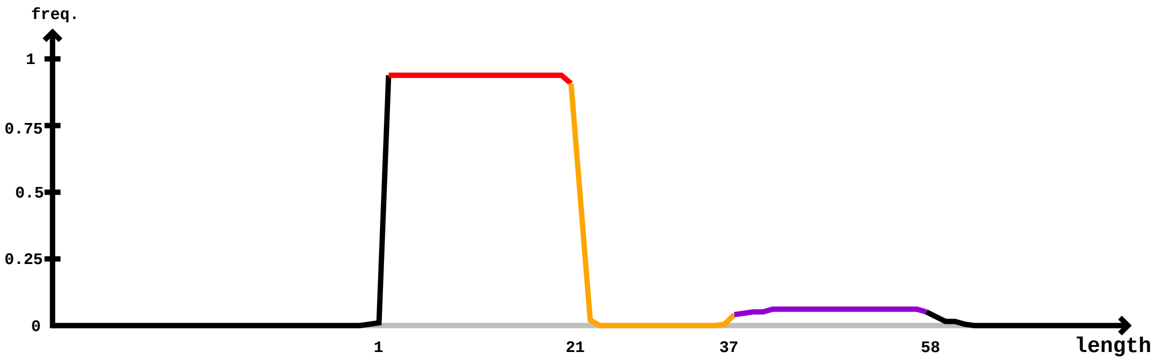

Mature Star

```
5'- ccuggggaggaagggauguacuggguuggcccaaaaguucguucggguuuuuccauuagcugguacagacaacccgaaugaacuuuuuggccaauucaauauuuccagc  -3'   obs
    ccuggggaggaagggauguacuggguuggcccaaaaguucguucggguuuuuccauuagcugguacagacaacccgaaugaacuuuuuggccaauucaauauuuccagc         exp
    (((.....)))...(((.(((.((((((((((.(((((((((((((((((((((((.....)))...))).))))))))))))))))).)))))))))).))).)))...  reads   mm   sample
    ...............................cAaaaaguucguucggguuuuu........................................................  1       1    seq
    ................................Aaaaaguucguucggguuuu.........................................................  1       1    seq
    .................................aaaaguucguucggguuuu.........................................................  5       0    seq
    .................................aaaaguucguucUgguuuuu........................................................  1       1    seq
    .................................aaaaguucguucggguuuuC........................................................  10      1    seq
    .................................aaaaguucguucggguuuuu........................................................  99      0    seq
    .................................aaaaguucguucggguuuuuc.......................................................  62      0    seq
    .................................aaaaguucguucggguuuuucc......................................................  1       0    seq
    .................................aaaaguucguucggguuuuucU......................................................  3       1    seq
    ..................................................................gaAaacccgaaugaacuuuuu......................  1       1    seq
    ....................................................................acaacccAaaugaacuuuuu.....................  1       1    seq
    ....................................................................aAaacccgaaugaacuuuuug....................  6       1    seq
    .....................................................................caacccgaaugaacuuuuug....................  1       0    seq
    ......................................................................aacccgaaugaacuuuuuggcc.................  1       0    seq
    ........................................................................cccgaaugaacuuuuuggcU.................  1       1    seq
    ........................................................................cccgaaugaacuuuuuggccG................  1       1    seq
```
